# Effect of using HIV and infant feeding counselling cards on the quality of counselling provided to HIV positive mothers: a cluster randomized controlled trial

**DOI:** 10.1186/1746-4358-6-13

**Published:** 2011-09-26

**Authors:** Mary Katepa-Bwalya, Chipepo Kankasa, Olusegun Babaniyi, Seter Siziya

**Affiliations:** 1 World Health Organization, Lusaka, Zambia; 2 Department of Paediatrics & Child Health, University Teaching Hospital, Lusaka, Zambia; 3 Department of Community Medicine, School of Medicine, University of Zambia, Lusaka, Zambia

**Keywords:** infant feeding, breastfeeding, young children feeding, HIV, counselling cards

## Abstract

**Background:**

Counselling human immunodeficiency virus (HIV) positive mothers on safer infant and young child feeding (IYCF) options is an important component of programmes to prevent mother to child transmission of HIV, but the quality of counselling is often inadequate. The aim of this study was to determine the effect the World Health Organization HIV and infant feeding cards on the quality of counselling provided to HIV positive mothers by health workers about safer infant feeding options.

**Method:**

This was a un-blinded cluster-randomized controlled field trial in which 36 primary health facilities in Kafue and Lusaka districts in Zambia were randomized to intervention (IYCF counselling with counselling cards) or non- intervention arm (IYCF counselling without counselling cards). Counselling sessions with 10 HIV positive women attending each facility were observed and exit interviews were conducted by research assistants.

**Results:**

Totals of 180 women in the intervention group and 180 women in the control group were attended to by health care providers and interviewed upon exiting the health facility. The health care providers in the intervention facilities more often discussed the advantages of disclosing their HIV status to a household member (RR = 1.46, 95% CI [1.11, 1.92]); used visual aids in explaining the risk of HIV transmission through breast milk (RR = 4.65, 95% CI [2.28, 9.46]); and discussed the advantages and disadvantages of infant feeding options for HIV positive mothers (all p values < 0.05). The differences also included exploration of the home situation (p < 0.05); involving the partner in the process of choosing a feeding option (RR = 1.38, 95% CI [1.09, 1.75]); and exploring how the mother will manage to feed the baby when she is at work (RR = 2.82, 95% CI [1.70, 4.67]). The clients in the intervention group felt that the provider was more caring and understanding (RR = 1.81, 95% CI [1.19, 2.75]).

**Conclusion:**

The addition of counselling cards to the IYCF counselling session for HIV positive mothers were a valuable aid to counselling and significantly improved the quality of the counselling session.

## Background

Strategies that aim at reducing Mother to Child Transmission (MTCT) of the Human Immunodeficiency Virus (HIV) are the cornerstone in reducing the prevalence of HIV in children. Antenatal care (ANC) attendance in Zambia is high (94%) with more than 90% of women attending ANC services being tested for HIV [1]. With a high antenatal HIV prevalence, estimated at 16.4% in 2008, approximately 80,000 infants born annually in Zambia are at risk of acquiring HIV from their mothers. For the majority of mothers in sub-Saharan Africa, where both HIV prevalence and infant mortality are high, breastfeeding an infant is particularly important for child survival [2-4]. Exclusive breastfeeding has been shown to have a lower risk of HIV transmission as compared to mixed feeding [5-7]. According to the Zambia Demographic Health Surveys (ZDHS) of 2002 and 2007, the six months exclusive breastfeeding rates increased from 41 to 60% respectively [8]. Replacement feeding remains elusive for the majority who do not fulfil the AFASS (affordable, feasible, acceptable, sustainable and safe) criteria [9]. Given the risk of transmission of HIV through breast milk, efforts to make breastmilk as safe as possible remains an important aspect in the prevention of MTCT (PMTCT) in Zambia. Counselling HIV positive mothers so that they may make informed choices on safer infant feeding options is an important component of national programmes to prevent MTCT. Zambia adopted and adapted the 2003 World Health Organization (WHO) recommendations [9] on infant feeding and these were part of the PMTCT guidelines until November 2010 when Zambia adopted the new recommendations [10]. Research in South Africa and Brazil showed that the quality of counselling provided to HIV positive mothers on safer infant feeding options was inadequate [11-14]. This was despite the fact that health providers had good general counselling skills and received training on HIV and infant feeding counselling. In an effort to improve the counselling of HIV positive mothers, WHO has developed counselling cards to be used as job aids, to complement the HIV and infant feeding counselling training. Job aids are visual images with messages which give step by step guidance to the provider and have been shown to improve client understanding [15,16]. The study aimed to determine the effect of using HIV and infant feeding counselling cards on the quality of counselling provided to HIV positive mothers about safer feeding options. We report comparisons of processes and outcomes of counselling between health workers in the intervention (with infant feeding counselling cards) and non-intervention (without infant feeding counselling cards) arms.

## Methods

### Study area

The study took place in primary health facilities in Lusaka and Kafue districts of Lusaka Province between April and June 2007. The health facilities in the two districts all offer prevention of MTCT and infant feeding counselling to mothers who are HIV positive as part of the focused antenatal care (FANC) services.

### Sample size

It was hypothesized that the use of HIV and infant feeding counselling cards as job aids by health workers offering infant feeding options to HIV positive mothers would result in a 40% increase in the mothers who would receive appropriate infant feeding counselling. We obtained 18 health facilities (clusters) in the intervention group and another 18 health facilities in the control group. A total of 10 women were recruited from each health facility, giving 180 women in the intervention group and another 180 women in the control group.

$$\text{c}=1+\left\{{\left({\text{z}}_{1}+{\text{z}}_{2}\right)}^{2}\left[2\text{p}\left(1-\text{p}\right)∕\text{n}+{\text{k}}^{2}\left({{\text{p}}_{1}}^{2}+{{\text{p}}_{2}}^{2}\right)\right]\;\right\}∕{\left({\text{p}}_{2}-{\text{p}}_{1}\right)}^{2}$$

Where

c = number of clusters required per group

p_1 _= proportion in intervention group

p_2 _= proportion in control group

p = (p_1 _+ p_2_)/2

z_1 _= percentage point for error

z_2 _= percentage point for error

n = number of individuals in each cluster

k = coefficient of variation of proportions (risks) among clusters in each group (*which is estimated from the range of outcomes across clusters*)

### Sampling

The health workers in the Maternal and Child Health (MCH) unit who normally offer FANC and infant feeding counselling services at the selected health facilities were recruited to participate in the study. The health workers from the randomly selected intervention sites were trained to use the counselling cards. The mothers who were known to be HIV positive were sequentially enrolled so long as they agreed to participate in the study.

### Study design

**Figure 1:**
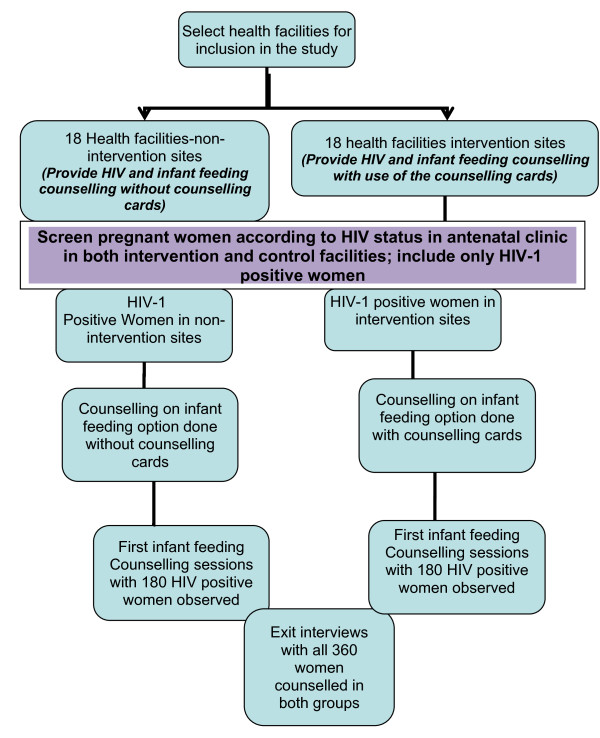
**Evaluation of HIV and Infant Feeding Counselling Cards: Synopsis of the study**.

Figure 1 (Evaluation of HIV and Infant Feeding Counselling Cards: Synopsis of the Study) shows the flow of participants in the study. Thirty-six (36) health facilities in Kafue and Lusaka districts were randomized into intervention and non-intervention sites. The grouping and randomisation of health facilities was done in WHO headquarters, Geneva and provided to the Principal Investigator (PI) two weeks prior to the orientation of health workers from the intervention sites. The randomization took into consideration the health facility's catchment population and the distance from the district health management offices. Half the health facilities were randomized to intervention sites and their health workers were oriented in the use of the HIV and infant feeding counselling cards, and the other half were randomized to non-intervention sites.

Twenty-seven health workers from the intervention sites were oriented through a three day workshop before the implementation of the intervention. They had previously been trained in HIV and infant feeding counselling as part of the training in prevention of MTCT. The counselling cards were used as job aids to complement this training. They were then followed and given supervisory support over a period of 6 to 12 weeks by three experienced supervisors, the PI and co-PI. Twenty experienced research assistants were oriented on the use of the data collecting tools (observation checklist and client exit questionnaire) over a day. During the orientation, the research assistants pre-tested the data collecting tools and both the clients' and providers' informed consent forms. The trained research assistants collected data from the health workers through an observation checklist and from the mothers through a client exit interview. Data was collected on the counselling sessions as well as the services being offered by the health facilities. The research assistants visited assigned health centres and observed at least two counselling sessions per day over a five day period. The target populations were pregnant women who were HIV positive and attending ANC clinic, and health workers providing HIV and infant feeding counselling, at both the intervention and non-intervention sites.

### Ethics

Ethical clearance was obtained from the University of Zambia Biomedical Research Ethics Committee (Assurance No. FWA00000338, IRB0000774). Permission to conduct the study was obtained from the Lusaka Provincial Health Office, Lusaka District Health Management Team (DHMT) and Kafue DHMT. Written consent was obtained from the health providers and mothers who agreed to participate in the study.

### Data collection

Data were collected using an observation check-list during a counselling session and a client exit questionnaire which was administered immediately after the infant feeding counselling session within the health facility. The observation check-list was used to assess the health worker's communication and counselling skills, and if questions related to understanding the risks of HIV transmission, decision-making process for choosing feeding options and exploration of home situation questions were asked. The client exit questionnaire covered some basic characteristics of the study population, the mother's understanding of the infant feeding counselling session, and how she assessed the provider in terms of counselling skills.

### Data management and analysis

Data was entered and cleaned using Epi-Data and exported to SPSS for analysis. Analysis was done using Complex samples program. We used the 95% confidence interval for the mean difference to compare means and the 95% confidence interval (CI) for the odds ratio (OR) to compare proportions at baseline. Proportions were compared using the 95% CI for the relative risk (RR). We also investigated whether education and occupation confounded the significant associations between the exposure (intervention/control) and various outcomes. Stratified analyses were conducted for relationships that were identified to be confounded by education and occupation.

## Results

There were 360 mothers who were counselled by the providers; 180 women in the intervention site and 180 women in the non-intervention site. All the women who were counselled by the providers were also interviewed upon exiting the counselling session. There were 1 to 2 providers observed for each health facility.

### Characteristics of the study population

Table 1 shows the distributions of characteristics between the intervention and control groups. The study groups were similar in terms of the factors: age of the respondent, marital status, number of living children, gestational age, and husband/partner accompanying respondent to clinic. However, more women in the intervention (18%) than control (7%) groups completed secondary, college or university levels of education and more women in the intervention (12%) than control (3%) groups were in salaried jobs or were self-employed professionals.

**Table 1:** Characteristics of the study population

Factor	Intervention Total = 180* n (%)	Control Total = 180* n (%)	Estimate (95%CI^#^) ^a^Mean difference (95%CI) ^b^Odds Ratio (95%CI)
Age of the respondent (years) [Mean (95%CI)]			
	Total = 178	Total = 180	
	27.1 (26.3, 27.8)	26.8 (25.9, 27.7)	0.27 (-0.88, 1.42)^a^
Marital status			
Married	159 (89.3)	150 (83.8)	1.62 (0.73, 3.58)^b^
Education			
Completed	32 (18.1)	13 (7.2)	2.84 (1.21, 6.65)^b^
secondary/			
some college or			
university			
Occupation			
Salaried job	22 (12.3)	6 (3.3)	4.06 (1.73, 9.54)^b^
or self-employed			
professional			
Number of living children			
> 2	57 (33.3)	64 (37.7)	0.81 (0.49, 1.33)^b^
Gestational age (weeks)	Total = 134	Total = 129	0.77 (-1.39, 2.93)^a^
[Mean (95%CI)]	31.2 (29.6, 32.8)	30.4 (28.9, 32.0)	
Husband/partner accompanied respondent to clinic			
Yes	8 (4.6)	5 (2.9)	1.61 (0.26, 9.80)^b^
No	167 (95.4)	168 (97.1)	

* *Totals may not add up due to missing information*

^# ^95% Confidence Interval

### Explaining the risks of HIV transmission

The research assistants observed that even though more health workers in the intervention site (66%) than in the control site (22%) discussed the risk of transmission of HIV (p < 0.001), the perception of the level of risk of HIV transmission through breastfeeding was not significantly different between the intervention and control groups.

### General counselling skills and decision-making process for choosing feeding option

Counsellors in the intervention group provided longer counselling sessions, more non-verbal communication, used more open-ended questions, and had better quality of counselling than counsellors in the control group. These results are shown in Table 2.

**Table 2:** General counselling skills and decision-making process for choosing feeding option

Factor	Intervention Total = 180* n (%)	Control Total = 180* n (%)	Relative Risk (95%CI) ^#^
Time taken for counselling sessions (minutes)			
Mean (95% CI)	Total = 164 35.6 (32.7, 38.5)	Total = 161 30.9 (27.4, 34.4)	2.2(0.3, 9.0)^a^
Provider established rapport	179 (100)	175 (97.8)	1.02 (1.00, 1.05)^b^
Provider listened effectively	171 (96.6)	158 (88.8)	1.09 (0.99, 1.20)
Provider used helpful non-verbal communication	168 (94.4)	142 (79.3)	1.19 (1.02, 1.39)
Provider used open-ended questions	160 (90.4)	129 (72.1)	1.25 (1.04, 1.52)
Provider used words that sound judging	30 (17.4)	19 (10.7)	1.63 (0.73, 3.68)
Provider used visual aids in explaining risk of HIV transfer through breast milk	141 (79.2)	30 (17.0)	4.65 (2.28, 9.46)
Provider discussed possible advantages for informing partner of her HIV status	166 (92.7)	146 (82.0)	1.13 (0.99, 1.29)
Provider discussed possible advantages for informing anyone else living in the household of her HIV status	152 (84.9)	103 (58.2)	1.46 (1.11, 1.92)
Partner involvement in infant feeding decisions discussed	161 (91.0)	116 (65.9)	1.38 (1.09, 1.75)
Provider checked mother's understanding of her feeding choice	166 (93.8)	129 (72.9)	1.29 (1.08, 1.53)
Provider performance with regards to quality of counselling was excellent	123 (69.5)	69 (38.5)	1.80 (1.09, 2.97)

* *Totals may not add up due to missing information*

^# ^95% Confidence Interval

^a ^Mean difference (95% Confidence Interval)

^b ^Confidence Interval includes 1

During the counselling sessions, the research assistants observed that health providers in the intervention group were about four times more likely to be rated as "excellent" with regard to quality of family notification than those in the control group. Furthermore, health providers in the intervention group were 46% more likely to discuss possible advantages of informing someone other than her partner living in the household of her HIV status than those in the control group. However, there were no significant differences between the two study groups in the proportion of health providers discussing the possible advantages of informing her partner of her HIV status.

The decision making process for choosing a feeding option was different between groups (Table 2), with 91% of health providers in the intervention and 66% of health providers in the control group having discussed partner involvement in infant feeding decisions. Furthermore, 94% of health workers in the intervention and 73% of health workers in the control group checked mothers' understanding of their feeding choices.

From the exit interviews, more mothers in the intervention (71%) than control (48%) groups reported that the length of consultation with the clinical staff was of the right amount of time. Clients in the intervention group were 91% less likely to be hurried in providing services as compared to those in the control group.

### Discussion of advantages and disadvantages of infant feeding options

Significantly (p < 0.05) more health providers in the intervention compared to control group discussed the advantages and disadvantages of the different feeding options (Table 3). However, the proportions of health providers discussing risks of death from formula feeding versus exclusive breastfeeding were not significantly different in the two groups. Overall, health providers in the intervention group were about five times more likely to be graded by research assistants as excellent in performance with respect to the quality of discussing advantages and disadvantages for infant feeding options compared to those in the control group.

**Table 3:** Discussion of advantages and disadvantages of infant feeding options

Provider discussed advantages and disadvantages for the following infant feeding options	Intervention	Control	Relative Risk (95%CI) ^#^
	Total = 180*	Total = 180*	
	n (%)	n (%)	
*Exclusive breastfeeding for the first 6 months followed by cessation of breastfeeding*			
Advantages			
Yes	171 (100)	168 (94.9)	1.05 (1.02, 1.09)
No	0 (0)	9 (5.1)	
Disadvantages			
Yes	167 (98.8)	143 (84.1)	1.18 (1.05, 1.32)
No	2 (1.2)	27 (15.9)	
*Formula*			
Advantages			
Yes	163 (95.9)	137 (78.3)	1.23 (1.02, 1.47)
No	7 (4.1)	38 (21.7)	
Disadvantages			
Yes	160 (94.7)	127 (73.8)	1.28 (1.04, 1.58)
No	9 (5.3)	45 (26.2)	
*Expressed and heat treated breast milk*			
Advantages			
Yes	127 (77.9)	12 (7.9)	9.87 (4.78, 20.36)
No	36 (22.1)	140 (92.1)	
Disadvantages			
Yes	124 (76.5)	10 (6.8)	11.33 (4.90, 26.21)
No	38 (23.5)	138 (93.2)	
Mentioned risk of acquiring pneumonia for a baby on formula	133 (75.6)	51 (28.7)	2.64 (1.53, 4.55)
Mentioned risk of acquiring diarrhoea for a baby on formula	163 (92.6)	138 (77.5)	1.20 (1.01, 1.41)
Portrayed risks of death on formula higher than exclusive breastfeeding	94 (55.3)	73 (45.3)	1.22 (0.76, 1.95)
Health provider performance with regard to the quality of discussing advantages and disadvantages of infant feeding option was excellent	83 (46.6)	16 (8.9)	5.22 (2.19, 12.44)

* *Totals may not add up due to missing information*

^# ^95% Confidence Interval

### Exploration of home and family situation regarding the formula option

The health providers in the intervention group were 48% more likely to explore the family and home situation in eliciting mothers' response about the feasibility to formula feed; 84% more likely to inquire if client has money to buy formula or other animal milk, or to pay for transport to collect milk regularly; about two times more likely to inquire if client has access to adequate supplies of water and fuel; about two times more likely to inquire whether a client has a fridge; and about three times more likely to discuss how the mother would feed the infant at night than those in the control group (Table 4).

**Table 4:** Exploration of home and family situation regarding the formula option

Factor	Intervention Total = 180* n (%)	Control Total = 180* n (%)	Relative Risk (95%CI) ^#^
Provider elicited mother's response about the feasibility to formula feed	159 (89.3)	108 (60.3)	1.48 (1.13, 1.94)
Provider inquired if mother has money to buy formula or to pay for transport to collect milk regularly	156 (87.6)	85 (47.8)	1.84 (1.41, 2.39)
Provider inquired if client has access to adequate supplies of water and fuel	151 (84.8)	63 (36.2)	2.34 (1.50, 3.67)
Provider inquired whether client has a fridge	103 (57.5)	50 (28.1)	2.05 (1.13, 3.70)
Provider discussed how the mother would feed the infant at night	150 (83.8)	53 (30.1)	2.78 (1.80, 4.30)

* *Totals may not add up due to missing information*

^# ^95% Confidence Interval

### Supporting the mothers who chose the exclusive breastfeeding option

Health providers in the intervention (97%) and control (94%) groups checked the mothers' understanding of exclusive breastfeeding with no significant difference between the two study groups. The proportion of health providers mentioning cracked nipples as a condition requiring that mothers should come back immediately to the clinic were not significantly different between the groups. However, health providers in the intervention group were about three times more likely to ask how the mother will manage to feed the baby when at work or at school away from home during the day, three times more likely to check mothers' understanding about positioning and attachment, and about two times more likely to explain to mothers which conditions require that they should come back immediately to the clinic (Table 5).

**Table 5:** Supporting the mothers who chose the exclusive breastfeeding option

Factor	Intervention Total = 155* n (%)	Control Total = 160* n (%)	Relative Risk (95%CI) ^#^
Provider checked mother's understanding of EBF	150 (96.8)	150 (93.8)	1.03 (0.96, 1.11)
Provider asked how the mother will manage to feed the baby when away from home during the day	124 (81.0)	46 (28.7)	2.82 (1.70, 4.67)
Provider checked mother's understanding about positioning and attachment	126 (81.8)	49 (30.6)	2.67 (1.54, 4.64)
Provider explained conditions for which the mother should come back to the clinic immediately	126 (85.1)	78 (50.3)	1.69 (1.14, 2.50)
Provider mentioned cracked nipples as a condition for which the mother should come back to the clinic immediately	123 (96.1)	84 (95.5)	1.07 (0.62, 1.84)

* Totals may not add up due to missing information

^# ^95% Confidence Interval

### Stratified analyses

Education was identified as a confounder in the relationship between the exposure 'Intervention/Control' and the following outcomes: 'Provider inquired if client had access to adequate supplies of water and fuel', 'Provider discussed advantages for expressed and treated breastmilk', 'Provider discussed disadvantages for expressed and heat treated breastmilk', 'Provider discussed disadvantages for expressed and heat treated breastmilk', and 'Provider asked mothers how they would manage to feed the baby when at work or at school (away from home during the day)'. We thus stratified the analysis by educational level. Among the less educated clients, providers in the intervention group were about 3 times more likely to inquire if clients had access to adequate supplies of water and fuel, about 10 times more likely to discuss advantages of expressed and treated breast milk, about 12 times more likely to discuss disadvantages of expressed and heat treated breast milk, and about 3 times more likely to ask mothers how they would manage to feed the baby when at work or at school (away from home during the day) compared to providers in the control group. However, among the more educated clients, no significant associations were observed.

Occupation was identified as a confounder in the relationship between the exposure 'Intervention/Control' on one hand and the outcomes: 'Provider discussed advantages and disadvantages for expressed and heat treated breast milk' and 'Provider asked how the mother will manage to feed the baby when at work or at school (away from home during the day) on the other'. However, no significant associations were observed after stratifying the analysis by type of occupation.

## Discussion

With the high HIV prevalence in pregnant women, MTCT remains a significant challenge in Zambia. Currently 65% of HIV positive women attending ANC receive antiretroviral prophylaxis as part of a comprehensive PMTCT programme. As the programme of prevention of MTCT is scaled-up, it is important to invest efforts at all points in time when the child gets infected: in-utero, at delivery and post-partum. This study highlighted the importance of improving the quality of IYCF counselling sessions so as to provide the mother and her family a better chance to make the appropriate choice according to her own situation.

There were no significant differences between the intervention and control groups in most socio-economic characteristics except educational level and occupation. This may have an effect on the knowledge and health-care seeking practices between the two groups. It has been shown that those with higher education will tend to utilize the health services more and their health care practices will be better than those with less education. Knowledge on the risk and prevention of MTCT of HIV has been shown to increase with level of education and wealth quintile [8].

Discussion on the risk of transmission of HIV through breast milk was done well in both the intervention and non-intervention sites. This is not surprising as the general awareness of Acquired Immune Deficiency Syndrome (AIDS) is universal (99%) among all subgroups of women and men regardless of their background characteristics [8]. In the same report, 85% of women recognize that HIV can be transmitted through breastfeeding. The knowledge of strategies to reduce the risk of transmission of HIV through breastmilk by taking special drugs is still inadequate, with almost two thirds (63%) of the women being knowledgeable about this. With the use of counselling cards, it was observed that the providers gave better explanation of the risks of transmission of HIV. Even though the counsellors provided a better explanation of the risk of transmission of HIV, this was not the case in the client exit interview, where the understanding of the level of risk of transmission of HIV through breastmilk and infant feeding options was not significantly different between the intervention and control groups. It could be a reflection that the knowledge of risk of HIV was very well understood and the providers in each arm were able to give similar explanation. It could also be that the clients had access to other common sources of information like the radio, and as such there would be no difference in the understanding between the groups. In a study done in two districts in Zambia, (Kafue and Mazabuka), the most common source of information on HIV and infant feeding was the nurses, especially during FANC and under-five clinic visits when health talks are given to the caretakers [17].

Health workers are the most common source of information on infant and young child feeding, especially messages on breastfeeding. This has implications on the knowledge and skills that the health workers will pass on to the mothers. Health workers need to be knowledgeable about the feeding options in order to assist a mother to make better decisions on how best she can feed her infant. In settings where knowledge of feeding options is inadequate, the health providers tend to be stressed and are not too sure what to tell the mother. Research done in several countries reveals that the information about infant feeding provided to HIV-positive mothers with exposed children was inadequate, and this could jeopardise the prevention of MTCT of HIV [11-14]. Further studies done in Tanzania revealed that there were high levels of distress and frustration among the nurse counsellors as they found themselves unable to give qualified and relevant advice to HIV-positive mothers [18]. Studies conducted in South Africa and India have shown that infant feeding counselling is often inadequate and of poor quality, and in many instances not availed to the mothers who need it [11,19]. Improving the knowledge and skills of the health worker on infant and young child feeding is important in trying to address the issue of post-partum transmission of HIV [17]. The advantages and disadvantages of all the feeding options were outlined systematically in the intervention group. The counselling card was a job aid that assisted the counsellors to address all the feeding options without the need to memorise the contents and hence risk not thoroughly discussing an option. Job aids improve counselling sessions by standardising the messages delivered and systematically addressing topics step by step [15,16]. Messages that certain health workers give during infant feeding counselling sessions are influenced by their beliefs and perceptions and are sometimes different from the WHO recommendations [20]. Local adaptation to job aids is important, as a socially and culturally acceptable integrated set of infant feeding counselling tools enhance counselling sessions [21]. The WHO counselling cards were well accepted in the intervention site and there was no reported difficulty in their use by the providers and clients. All the health workers had been conducting infant feeding counselling previously and the cards were an additional aid to their counselling session. A 12-member team of experts had made minor adaptations to the cards according to the recommendations prevailing in Zambia then. Of note was that Step 2 was rearranged to reflect breastfeeding as the first option discussed with the client, followed by the commercial infant formula option. Modified cow's milk and the other options were discussed only when a client requested for the option. When informed choice on infant feeding methods is promoted, women's decisions might still be compromised by the advice given, due to some options not being accurately explained by workers [22]. Health workers' knowledge and imparting that information to mothers being counselled is thus very important.

Exploring the home situation and environment are important aspects of trying to see if the mother can meet AFASS to use formula. In addition, male involvement in child health is very low, especially when the child is young. This is a source of worry, as the aspect of home support for the chosen feeding option becomes questionable without the father being involved in terms of financial as well as emotional support. For the mother who chooses to use formula, support of family is very important if she is to do it successfully. In a culture where social expectations are to breastfeed, and where the father, relatives and the community are part of decision making on infant feeding, there is usually a gap between an intention to formula-feed and the actual infant feeding practice [23]. Mothers were more likely to practice mixed feeding, especially if there was no family support. A study done in Uganda found that women who successfully adhered to replacement feeding had family support [24]. Adherence to chosen feeding option is better with partner support than without it. In a study done in KwaZulu, Natal, Bland et al [25] found that adherence to feeding intention among HIV-infected women was higher in those who chose to exclusively breastfeed than those who chose replacement feeding.

The health providers in the intervention group were observed to spend significantly more time in the counselling sessions and the general counselling skills were better. This was further reflected in the exit interview where the clients seemed to appreciate the time and counselling skills of the providers in the intervention sites. With the addition of counselling cards, the counselling session would actually be more involving for the client and more appreciated. Having additional visual tools to aid the counsellor also added value to the counselling session. In the current study, the use of IYCF counselling cards clearly showed that the quality of counselling improved. With the aid of the card, the health workers were able to go through the process of counselling more systematically and importantly were able to talk about the home situation and involvement of the partner. In the intervention sites, the provider was perceived to be more caring and understanding. This is important in the follow-up of the mother-child pair, as the client is more likely to come back to the provider who seemed more concerned than one who appeared unconcerned with the client. The inadequate utilization of health services has been attributed to staff attitude in some instances. This is of concern, as the under-five clinic visits are important in encouraging and supporting the mother with her chosen feeding option. In addition, the infant will get tested at six weeks so that further services for those found to be HIV infected can be availed to them. A common challenge for most health providers is perceived increase in time spent with the client when there is added counselling and in this particular instance, with added counselling cards. In this study, there was a significant difference in time spent with provider, with the majority of clients in the intervention group saying that they spent the right amount of time with the health provider. Most often the clients are hurried through a session without clearing some misconceptions or misunderstandings they may have, and may end up practicing the wrong thing.

### Limitations

The study results may not be generalized to rural settings as the study was done in urban and peri-urban areas. The presence of an observer may have influenced the counselling session, but we are unable to determine its magnitude and direction.

## Conclusion

The counselling skills were better in the intervention group. The counselling cards made the counsellors go through the feeding options and the home situation more systematically as compared to the non-intervention sites. IYCF counselling cards improved the quality of counselling sessions. Even with the adoption of the new 2010 WHO recommendations on HIV and infant feeding [26], counselling will still be important in promoting exclusive breastfeeding, not only among HIV positive mothers, but other mothers as well. We recommend that IYCF counselling cards should be used in all counselling sessions to improve the quality of the counselling sessions, and health workers should be oriented in the use of the adapted IYCF counselling cards.

## List of abbreviations

AFASS: Affordable, feasible, acceptable, sustainable, safe; AIDS: Acquired Immune Deficiency Syndrome; ANC: Antenatal care; DHMT: District Health Management Team; FANC: Focused antenatal care; HIV: Human Immunodeficiency Virus; IYCF: Infant and young child feeding; MCH: Maternal and child health; MTCT: Mother to child transmission of HIV; PMTCT: Prevention of mother to child transmission of HIV; WHO: World Health Organization; ZDHS: Zambia Demographic Health Survey.

## Competing interests

The authors declare that they have no competing interests.

## Authors' contributions

MKB took part in proposal writing, conducted the study and participated in the drafting of the manuscript; CK was involved in proposal writing and conducted the study and SS conducted the analysis and took part in the drafting of the manuscript. CK, SS, and OB critically reviewed draft versions of the manuscript. All authors read and approved the final manuscript.

## Authors' information

MKB is a Paediatrician, public health specialist and researcher currently works as the National Profession Officer for Child and Adolescent Health at the World Health Organization, Zambia country office. She is also the WHO focal person for infant and young child feeding; CK is a Consultant Paediatrician, lecturer and researcher, currently the Director of the Paediatric Centre of Excellence for Paediatric HIV/AIDS and PI for the UTH HIV and AIDS programme (UTH-HAP) at the University Teaching Hospital; OB is the WHO Representative in Zambia, an epidemiologist, public health specialist and researcher; SS is a Professor of medical biostatistics and researcher currently teaches in the Department of Community Medicine in the School of Medicine of the University of Zambia.
